# *Bacillus subtilis* B579 Controls Cucumber Fusarium Wilt by Improving Rhizosphere Microbial Community

**DOI:** 10.3390/microorganisms13061382

**Published:** 2025-06-13

**Authors:** Zongqiang Fan, Jinghan Feng, Lixue Zheng, Yanru Chen, Minglei Wang, Xiangqian Peng, Shuo Wang, Fang Chen

**Affiliations:** 1 School of Pharmaceutical Sciences and Food Engineering, Liaocheng University, Liaocheng 252000, China; fzq19990323@163.com (Z.F.); fengjinghan21@126.com (J.F.); zhenglx2199@163.com (L.Z.); 18354153187@163.com (Y.C.); w1357924690@163.com (M.W.); pxq8848@126.com (X.P.); wangshuo@lcu.edu.cn (S.W.); 2 Shandong Key Laboratory of Applied Technology for Protein and Peptide Drugs, Liaocheng University, Liaocheng 252000, China; 3 State Key Laboratory of Macromolecular Drugs and Large-Scale Preparation, Liaocheng University, Liaocheng 252000, China

**Keywords:** rhizosphere microorganism, *B. subtilis B579*, cucumber Fusarium wilt, biological control, soil environment

## Abstract

With continuous improvements in people’s environmental awareness, biological control agents have garnered considerable attention owing to their advantageous impacts on improving soil fertility and alleviating plant diseases. *Bacillus subtilis* (*B. subtilis*) B579, isolated from the rhizosphere soil of cucumber, has effectively suppressed the growth of pathogenic *Fusarium oxysporum*. Our study investigates the effects of *B. subtilis* B579 on the properties of the rhizosphere soil (its physicochemical properties and enzymatic activities) and microbial community of cucumber under *Fusarium oxysporum* infection. An amplicon sequencing analysis of the microorganisms in the rhizosphere soil was conducted, and the soil’s properties were measured. The findings demonstrated that *B. subtilis* B579 exhibited 73.68% efficacy in controlling cucumber Fusarium wilt disease. B579 pretreatment substantially increased the bacterial and fungi diversity and improved the soil’s physicochemical properties (pH level and OC, TN, TP, AK, and AP contents) and enzyme activities, especially those of urease and alkaline phosphatase, which exhibited significant increases of 77.22% and 64.77%, respectively, in comparison to those under the pathogen treatment. Furthermore, the utilization of B579 reduced the abundance of *Fusarium* while simultaneously increasing the abundance of beneficial groups, including the *Bacillus*, *Paenibacillu*s, *Sphingomonas*, *Pseudomonas*, *Microbacterium*, *Mortierella*, and *Trichoderma* genera. The RDA showed that the abundance of *Bacillus*, *Paenibacillus*, *Sphingomonas*, and *Mortierella* in the rhizosphere showed positive correlations with most of the soil properties, whereas *Fusarium* abundance was negatively correlated with most of the soil’s properties. This study provides novel insights into the disease suppression mechanisms of *Bacillus subtilis* B579, laying the theoretical foundation for its development as a biocontrol agent.

## 1. Introduction

Cucumber *(Cucumis sativus* L.), a member of the *Cucurbitaceae* family, ranks among the top in the world in terms of its planting scale and yield [1]. Its fruits are rich in nutrients such as protein, calcium, lignans, cucurbitacin, and ascorbic acid, making it popular among the general public [2]. Cucumber Fusarium wilt, caused by *Fusarium oxysporum* f. sp. cucumerinum (FOC), may occur at any stage during the cucumber’s development, resulting in plant wilting, death, and ultimately yield reductions [3]. Control of Fusarium wilt has traditionally relied on methods such as selective breeding, grafting, soil fumigation, and pesticide spraying, among others. However, these conventional approaches have certain limitations [4]. The methods of disease resistance breeding and grafting are considered secure approaches to controlling cucumber Fusarium wilt, but they can potentially influence certain crop characteristics, such as yield and taste [5,6]. Soil fumigation destroys the inherent ecological balance of the soil’s microorganisms, leading to a reduction in beneficial microorganisms, which in turn impairs crop development [7]. Although chemical fungicides act rapidly, their use may induce pathogen resistance and cause irreversible soil contamination [8]. Biological control methods have been widely used to manage Fusarium wilt in vegetables due to their safety, efficiency, and environmental protection [9].

Biological control agents (BCAs) are usually derived from rhizosphere bacteria or fungi, which have a strong ability to colonize the soil and can provide effective protection against pathogen invasion to plants [10]. As an extensively studied biological control agent, *Bacillus* has demonstrated significant potential in suppressing fungal pathogens. Recent studies have shown that *B. subtilis* Z-14 can inhibit the synthesis of the cell membrane and disrupt the energy metabolism in the pathogen’s mycelium, thereby protecting plants from *F. oxysporum* infection [11]. Studies showed that the incidence of Fusarium wilt was markedly reduced following exposure to *Bacillus velezensis* F9 in pathogen-challenged cucumber plants [12]. Yang et al. [13] reported that *Bacillus subtilis* 1JN2 could inhibit pathogenic mycelial growth and increase the abundance of beneficial microbial species, thus protecting the plant rhizosphere from pathogen-induced damage.

Microbial communities in the soil play a crucial role in maintaining soil health and enhancing the capacity of plants to resist diseases [14]. However, rhizosphere microbial communities are often shaped by farming practices, the host genotype, and the soil’s enzyme activity, among other factors, which are closely related to a plant’s disease suppression [15,16,17]. In past decades, microbial diversity was primarily determined through traditional culture and plate counting, phospholipid fatty acid analysis (PLFA), and denaturing gradient gel electrophoresis (PCR-DGGE) [18,19]. However, these methods are time-consuming and face difficulty in attaining the depth necessary to recognize more microorganisms [20]. The application of amplicon sequencing technology can efficiently sequence DNA fragments. It not only shortens the research time but also significantly improves the detection of non-dominant microbial communities [21,22]. To our knowledge, no previous analysis using amplicon sequencing technology to evaluate the effect of *B. subtilis* B579 on the microbial communities in the rhizosphere soil of cucumber has been conducted.

*Bacillus subtilis* B579 was initially screened and isolated from the rhizosphere of cucumber. In previous studies, we found that B579 had a strong ability to colonize the soil and exhibited potent antagonism against *Fusarium oxysporum* [23]. The principal objectives of this study were (1) to assess the efficacy of B579 in controlling Fusarium wilt in cucumber; (2) to assess how *B. subtilis* B579 modulated the cucumber’s rhizosphere microbiome under *F. oxysporum* challenge; and (3) to elucidate the mechanisms underlying fungal disease suppression in plants further.

## 2. Materials and Methods

### 2.1. Strain Culture

*B. subtilis* B579 (GenBank: EU221616.1) was cultured in a flask containing 30 mL of Luria–Bertani Broth (LB) medium. Following 16 h of cultivation at 180 rpm and 37 °C, 500 μL of the bacterial pre-culture (10^5^ CFU (colony forming units)/mL) was transferred into a new flask containing 50 mL of LB medium and cultured at 37 °C and 180 rpm for 24 h. The bacterial cells were harvested through centrifugation at 8000× *g* for 6 min, and the pellet was resuspended in sterile distilled water to a final concentration of 2 × 10^8^ CFU/mL for inoculation.

The pathogen, originally isolated from rotten cucumber roots and identified as *F*. *oxysporum* (GenBank: GCA_001702555.1), was cultured on potato dextrose agar (PDA) plates at 28 °C for 6 d. Subsequently, the plates’ surfaces were carefully rinsed with distilled water to prepare the spore suspensions. This pathogen exhibited high pathogenicity in our previous study.

### 2.2. The Pot Experimental Design

The pot experiment was carried out at the agricultural cultivation base of Liaocheng University (Liaocheng, China). The cucumber cultivar selected for this experiment was “Jinyou 2”, which is susceptible to Fusarium wilt. The seeds were surface-sterilized through immersion in 0.1% (*w*/*v*) potassium permanganate solution for 30 min. After sterilization, the seeds were thoroughly rinsed three times with sterile distilled water and then transferred onto sterile Petri dishes containing moist, sterile filter paper. Germination was carried out at 28 °C in darkness for 48 h. Germinated seeds with a normal morphology were sown into flowerpots (capacity: 10 L) filled with a mixed substrate (5:3:2, *v*/*v*, soil, vermiculite, and leaf compost). Prior to use, the substrate was piled into multiple rectangular mounds (10 m × 2 m × 0.2 m) in the greenhouse, watered to saturation, and covered with 0.06 mm white plastic film. To minimize heat loss, the edges of the film were sealed with soil. Temperature monitoring showed that the average soil temperature inside the film reached 61 °C at 11:00 and 74 °C at 13:00 during the disinfection period from 27 June to 27 July 2023. The mixed substrate was air-dried and then homogenized using a blender. The properties of the mixed substrate were as follows: pH, 7.51; TP, 1180 μg/g; TN, 1.36 g/kg; OC 34.45 g/kg; AK 82.37 mg/kg; OP: 546.88 μg/g. The cucumber plants were grown under the following conditions: 26 °C daytime/18 °C nighttime temperatures, natural light, and 75% relative humidity.

The inoculation treatment was performed once the second true leaf had fully developed. This experiment encompassed four treatments: (1) CK (cucumbers irrigated with distilled water (control)); (2) B579 (cucumbers irrigated with the B579 suspension); (3) B579 + FOC (BF) (cucumbers irrigated with the B579 suspension and subsequently with FOC); and (4) FOC (cucumbers irrigated with the FOC suspension). The inoculation process was primarily divided into two distinct stages. In the initial stage, 50 mL of B579 suspension (2 × 10^8^ CFU/mL) was introduced into the cucumber rhizosphere soil. After a 3-day interval, 50 mL of the pathogen spore suspension (1 × 10^8^ conidia/mL) was irrigated into the rhizosphere soil. In both stages, in cases where the B579 suspension or the pathogen spore solution was not required for the treatment, an equivalent volume of distilled water was utilized as a replacement. Suspensions were delivered to the rhizosphere soil at a depth of approximately 4 cm via a sterile syringe using a root irrigation method. Seedlings were arranged in a completely randomized block design with three replications for each treatment. Each pot contained three seedlings, totaling 72 plants per treatment. After 13 days post-pathogen inoculation, the disease severity was classified into one of five categories based on the symptoms induced by FOC: 0 for healthy plants, 1 for mild wilting in the cotyledons, 2 for etiolated cotyledons, 3 for etiolated and seriously distorted plants, and 4 for plant mortality. The disease severity index was calculated using the following equation: Disease index = [∑(rank × number of plants rated)/(number of plants investigated × highest rank)] × 100% [24].

### 2.3. Soil Collection and Analysis of Plant Growth 

On day 13 after FOC inoculation, we randomly selected three plants within each treatment. The surface soil was removed, and the plant roots were carefully uprooted and gently shaken to remove loosely attached soil. Using a 2 mm sieve, the tightly adhered rhizosphere soil was sieved and collected. One portion of each sample was utilized to determine the soil’s physicochemical properties and enzyme activities, whereas the remaining portion was used for DNA extraction.

Meanwhile, the sampled plants were rinsed with normal saline, and excess moisture was blotted using filter paper. The plant height, stem diameter, fresh weight, and dry weight of the cucumber seedlings were recorded.

### 2.4. Analysis of the Soil’s Physicochemical Properties 

The soil pH was measured using a potentiometric method. The total nitrogen (TN) content was analyzed using ultraviolet spectrophotometry following alkaline potassium persulfate digestion [25]. The organic carbon (OC) content was analyzed through the potassium dichromate oxidation method with sulfuric acid digestion [26]. The available potassium (AK) content was measured using the sodium tetraphenylboron turbidimetry method. The total phosphorus (TP) and organic phosphorus (OP) contents were determined through the Mo-Sb antispectrophotometric method, and the available phosphorus (AP) content was measured through the phosphomolybdenum blue spectrophotometric method [27]. The OC, AK, TP, OP, and AP contents were determined using the respective kits (Hefei Laier Biotechnology Co., Ltd., Hefei, China) following the manufacturer’s protocol.

### 2.5. Analysis of the Soil’s Enzyme Activity

The soil urease (UR) activity in the soil samples was measured using the indophenol blue colorimetric method (the phenol-sodium hypochlorite assay), based on quantification of the ammonia released from urea hydrolysis [28]. The absorbance at 625 nm was measured using a UV-3600i spectrophotometer (Shimadzu, Tokyo, Japan). The soil’s urease activity was expressed as the milligrams of NH_4_^+^-N produced per gram of soil per hour (mg NH_4_^+^-N/g h). The soil’s alkaline phosphatase (ALP) activity was determined by measuring the release of phenol from phenyldisodium phosphate hydrolysis using 4-aminoantipyrine as a chromogenic agent [29]. The absorbance of the developing solution was measured at a wavelength of 660 nm using the UV-3600i spectrophotometer (Shimadzu, Tokyo, Japan). The soil’s alkaline phosphatase activity was expressed as milligrams of phenolic released per gram of soil per hour (mg phenol/g h). The soil’s chitinase (CHI) activity was determined using a spectrophotometric assay with p-nitrophenyl N-acetyl-β-D-glucosaminide (pNP-NAG) as the substrate, by quantifying the amount of p-nitrophenol (pNP) released by enzymatic hydrolysis [30]. The absorbance at 410 nm was measured using the UV-3600i spectrophotometer (Shimadzu, Tokyo, Japan). Chitinase activity was expressed as micrograms of N-acetylglucosamine released per gram of soil per hour (μg NAG/g h). The activities of catalase (CAT) and β-glucosidase (β-GLU) were measured using the protocols described by Xue et al. [31] and Dick et al. [32], respectively.

### 2.6. DNA Extraction from the Soil

Total genomic DNA was extracted from 0.4 g soil samples employing a fecal genomic DNA extraction kit (Nanjing Jiancheng Bioengineering Institute, Nanjing, China) under the manufacturer’s instructions. The concentration of the extracted DNA was measured using a NanoPhotometer (Implen, Munich, Germany), and quality was assessed through electrophoresis. For the subsequent analyses, qualified samples were diluted to 1 ng µL^−1^ using nuclease-free water and stored at −20 °C.

### 2.7. PCR and Sequencing

For each sample, the V3-V4 region of the bacterial 16S rRNA gene was amplified using the primers 515F (5′-CCTAYGGGRBGCASCAG-3′) and 806R (5′-GGACTACNNGGGTATCTAAT-3′) [33]. To amplify the fungal ITS sequence, the primers ITS1F (5′-CTTGGTCATTTAGAGGAAGTAA-3′) and ITS2R (5′-GCTGCGTTCTTCATCGATGC-3′) were adopted [34]. The PCR reaction mixture comprised 15 µL of Phusion^®^ High-Fidelity PCR Master Mix (New England Biolabs, Ipswich, MA, USA), 1 µL of each primer (0.2 µM), 10 ng of template DNA, and ddH_2_O to a total volume of 20 µL. The amplification conditions consisted of initial denaturation at 98 °C for 1 min, followed by 30 cycles of denaturation at 98 °C for 10 s, annealing at 50 °C for 30 s, and elongation at 72 °C for 30 s and 72 °C for 5 min. All PCR reactions were performed in triplicate. Every PCR run included a negative control reaction. After quantification through electrophoresis, the PCR products were normalized to an equal molarity and purified through 2% agarose gel extraction in 1 × TAE buffer. The target bands were recovered through shearing, followed by purification of the PCR products using the PureLink™ Gel Extraction Kit (Thermo Fisher Scientific, Waltham, MA, USA). The sequencing libraries were generated using the CloneMiner™ II cDNA library building kit (Thermo Fisher Scientific, Waltham, MA, USA). Following library validation via Qubit quantification and quality testing, sequencing was performed on the Illumina NovaSeq6000 platform (Illumina Inc., San Diego, CA, USA). High-throughput sequencing was performed by Novogene Co., Ltd. (Beijing, China) on 16 November 2023.

### 2.8. Sequence Data Processing

Paired-end reads were assigned to soil samples according to their distinct barcodes and segmented by cutting off the barcode and the primer sequence using Cutadapt in Python (v3.3) [35]. The reads from each sample were merged to generate raw tags using FLASH (v1.2.11) [36]. The raw tags were then filtered to gain high-quality clean tags using Fastp (v0.23.1) (sequences with >1% expected errors per base were discarded, and records with an abundance below 0.0001% of total amplicons were filtered out) [37]. Combined with the SILVA database (16S) (https://www.arb-silva.de, accessed on 16 November 2023) and the UNITE database (ITS), (https://unite.ut.ee, accessed on 16 November 2023), the chimeric sequences were detected and removed to acquire effective tags using the UCHIME algorithm (http://www.drive5.com/usearch/manual/uchime_algo.html, accessed on 16 November 2023) [38]. For the effective tags, denoising was performed utilizing DADA2 on the QIIME2 (v202202) platform (https://qiime2.org/, accessed on 16 November 2023) to acquire a table of initial Amplicon Sequence Variants (ASVs) [39]. Species annotation was conducted using QIIME2 software, and the databases for bacteria and fungi were, respectively, the “SILVA database” (version 138.1) [40] and the “UNITE database” (version 9.0) [41]. Using vegan v2.5-6, all sample data were normalized against the sample with the minimal data volume as the baseline [42]. Normalized datasets were used to conduct the subsequent alpha and beta diversity analyses.

### 2.9. Statistical Analysis

SPSS version 20.0 (SPSS Inc., Chicago, IL, USA) was used for the statistical analysis, and significance was assigned at *p* < 0.05 using a one-way analysis of variance (ANOVA), followed by Tukey’s tests. Generation of the rarefaction curves and calculation of the alpha diversity were conducted using QIIME2 (v202202). The relative abundance of taxa was calculated using R (v4.1.2). Based on the Bray–Curtis distance, the principal coordinate analysis (PCoA) was used to examine the dissimilarities in the soil’s microbial communities. A clustering heatmap of the dominant microbial species was generated using the “pheatmap” package in R. A redundancy analysis (RDA) was applied to exploring the correlation between microbial communities and the soil’s physicochemical properties using the Canoco 5.0. R software (v4.0.3), and GraphPad Prism version 8 (San GraphPad Software, Diego, CA, USA) was adopted for mapping.

## 3. Results

### 3.1. The Disease Index

**Figure 1:**
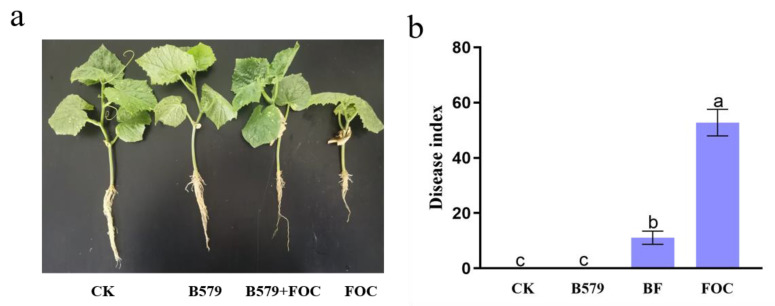
The growth status of the cucumber seedings (**a**) and the disease index (**b**) of Fusarium wilt under different treatments. Note: Values represent the mean ± SE (n = 3). CK (cucumbers, irrigated with distilled water (control)); B579 (cucumbers irrigated with the B579 suspension (2 × 10^8^ CFU/mL)); B579 + FOC (BF) (cucumbers irrigated with the B579 suspension for 3 days and subsequently with the FOC suspension (1 × 10^8^ conidia/mL)); FOC (cucumbers irrigated with the FOC suspension); values with the same letter do not differ significantly (*p* < 0.05) according to Tukey’s test.

To investigate the impact of B579 inoculation on the disease resistance of the cucumber seedlings, we assessed the plants’ infection status. The cucumber seedlings without FOC irrigation grew normally. After inoculating them with the pathogen, the plants displayed infection symptoms such as wilting and leaf yellowing under the FOC treatment conditions, with a disease index of 52.78%. However, pretreatment with B579 (BF) significantly reduced the disease index to 13.89% (Figure 1b), with less yellowing of the cucumber leaves observed (Figure 1a).

### 3.2. Plant Growth

**Figure 2:**
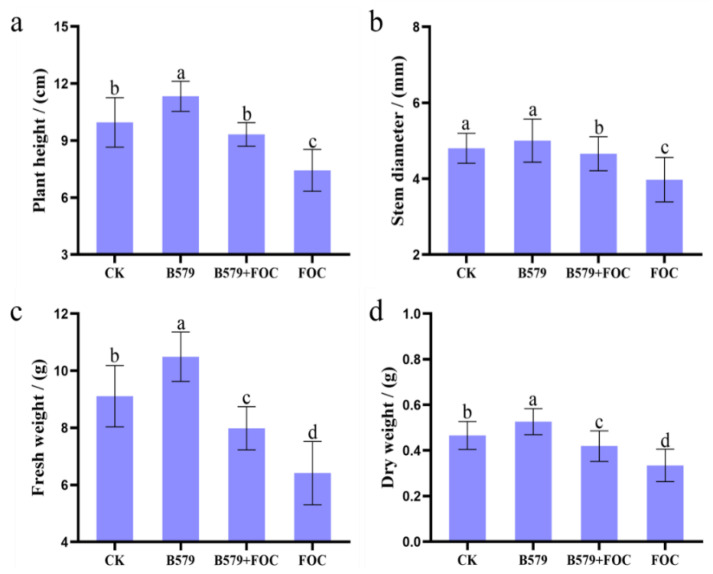
Growth indices of cucumber seedings in different treatments. (**a**) Plant height; (**b**) stem diameter; (**c**) fresh weight; (**d**) dry weight. CK (cucumbers irrigated with distilled water (control)); B579 (cucumbers irrigated with the B579 suspension (2 × 10^8^ CFU/mL)); B579 + FOC (BF) (cucumbers irrigated with the B579 suspension for 3 days and subsequently with the FOC suspension (1 × 10^8^ conidia/mL)); FOC (cucumbers irrigated with the FOC suspension). Note: Values represent the mean ± SE (n = 3). Values with the same letter do not differ significantly according to Tukey’s test (*p* < 0.05).

The effect of B579 inoculation on cucumber growth was assessed by measuring the plants’ height, stem diameter, fresh weight, and dry weight (Figure 2). All of the growth indices mentioned above showed active responses to the B579 treatment, and B579 inoculation promoted the cucumber seedlings’ growth. Compared with the control treatment, B579 treatment drastically increased the plants’ height by 13.87%, stem diameters by 4.17%, fresh weight by 15.15%, and dry weight by 12.77%. Interestingly, these four growth indicators in the cucumbers cultivated under the B579 + FOC conditions showed a notable rise relative to those under the FOC conditions (*p* < 0.05).

### 3.3. The Soil’s Physicochemical Properties and Enzyme Activities

To investigate whether B579 could effectively modify the rhizosphere soil environment to establish more favorable conditions, an analysis of the soil’s pH level and TP, TN, OC, AK, AP, and OP contents was performed. As presented in Table 1, the AK and AP contents increased the most following the B579 treatment, with values that were 29.05% and 32.59% higher, respectively, than those in the control treatment. Moreover, BF treatment obviously increased the pH level, as well as the content of TP, OC, AK, and AP, compared with these values under the FOC treatment. The most pronounced improvements were in the AK and AP contents, with increases of 23.45% and 45.60%, respectively.

Additionally, we quantified the activities of five soil enzymes: urease, alkaline phosphatase, catalase, chitinase, and β-glucosidase (Table 2). Compared with the control treatment, B579 treatment significantly improved the activities of urease (an increase of 17.13%), alkaline phosphatase (an increase of 37.96%), catalase (an increase of 20.58%), and β-glucosidase (an increase of 19.53%) (*p* < 0.05). Moreover, the activities of these four enzymes under the BF (BF + FOC) conditions were significantly elevated in comparison to those under the FOC conditions (*p* < 0.05). Notably, the urease and alkaline phosphatase activities increased by 77.22% and 64.77%, respectively.

### 3.4. Analysis of the Sequencing Results 

**Figure 3:**
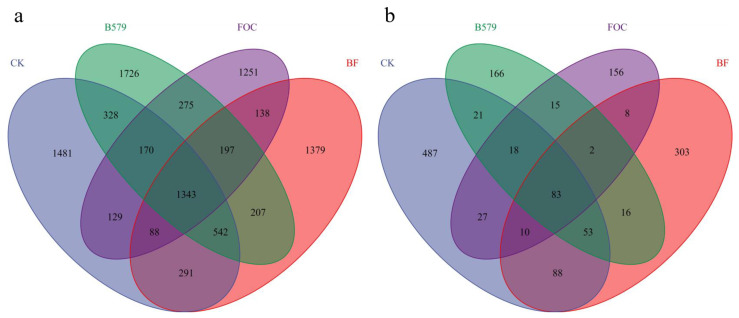
Venn diagram of the number of bacterial (**a**) and fungal (**b**) ASVs in different samples. CK (cucumbers irrigated with distilled water (control)); B579 (cucumbers irrigated with the B579 suspension (2 × 10^8^ CFU/mL)); B579 + FOC (BF) (cucumbers irrigated with the B579 suspension for 3 days and subsequently with the FOC suspension (1 × 10^8^ conidia/mL)); FOC (cucumbers irrigated with the FOC suspension).

**Figure 4:**
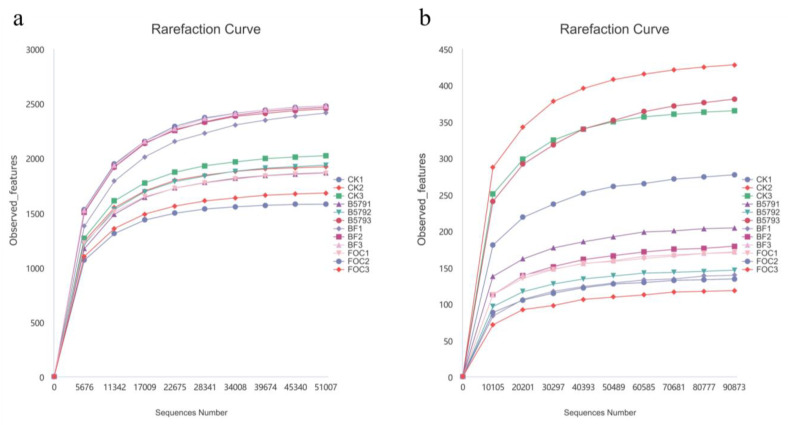
A rarefaction analysis for the soil samples collected from the four treatments. CK (cucumbers irrigated with distilled water (control)); B579 (cucumbers irrigated with the B579 suspension (2 × 10^8^ CFU/mL)); B579 + FOC (BF) (cucumbers irrigated with the B579 suspension for 3 days and subsequently with the FOC suspension (1 × 10^8^ conidia/mL)); FOC (cucumbers irrigated with the FOC suspension). (**a**) Bacteria; (**b**) fungi. The vertical axis shows the average number of observed features found after sampling the number of sequences shown on the horizontal axis.

In this study, a total of 959,170 16S rRNA gene sequences and 1,198,232 ITS gene sequences were acquired through splicing, filtering, and the removal of chimeras (Table 3). The mean number of bacterial sequences in each soil sample was 79,931 ± 13,498, while that for fungi was 99,853 ± 3922. Utilizing the DADA2 method for sequence noise reduction, a total of 9545 bacterial ASVs and 1453 fungal ASVs were detected (Figure 3). Venn diagrams revealed 1481, 1726, 1251, and 1379 unique bacterial ASVs in the CK, B579, BF, and FOC treatments, respectively, while 487, 166, 303 and, 156 unique ASVs were identified for fungi, respectively. Additionally, rarefaction curves were generated based on the amount of data extracted and the corresponding species numbers. As the amount of extracted data increased, we observed gradual flattening of the curves (for bacteria and fungi), suggesting that the sequencing data obtained were sufficient and reasonable (Figure 4).

Furthermore, the Shannon and Simpson indices were used to assess the community diversity, while the chao1 value was utilized to evaluate community richness (Table 4). The results demonstrated that the Shannon and Simpson indices for bacteria and fungi in the B579 + FOC-treated soil exhibited distinctly higher values than those in the FOC-treated soil (*p* < 0.05). Nevertheless, no remarkable differences were observed in these three indices for bacteria and fungi between the non-inoculated (CK) soil and the B579-treated soil.

### 3.5. PCoA

**Figure 5:**
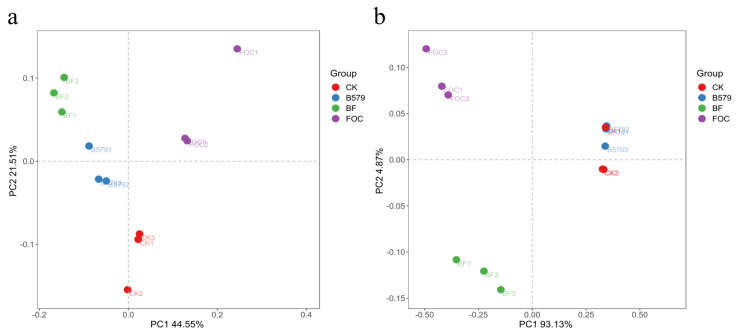
A principal coordinate analysis (PcoA) of the (**a**) bacterial and (**b**) fungal community from the cucumber rhizosphere soil samples. CK (cucumbers irrigated with distilled water (control)); B579 (cucumbers irrigated with B579 suspension (2 × 10^8^ CFU/mL)); B579 + FOC (BF) (cucumbers irrigated with B579 suspension for 3 days and subsequently with FOC suspension (1 × 10^8^ conidia/mL)); FOC (cucumbers irrigated with FOC suspension).

A principal coordinate analysis (PCoA) was performed to study the dissimilarity in the community structure among the soil samples. As shown in Figure 5, the first two principal components collectively explained 66.06% of the total variance in the bacterial community and 98.00% of the difference in the fungal community. Samples receiving the same treatment are closer together compared with the others in the PCoA plots. The bacterial community structure in the B579-treated soil closely resembled that in the B579 + FOC-treated soil, while the fungal community showed greater similarity to that in the non-inoculated soil. Moreover, the samples irrigated with FOC solely showed a clear difference from those irrigated with distilled water in terms of their bacterial and fungal community structures.

### 3.6. Analysis of the Soil’s Microbial Composition 

**Figure 6:**
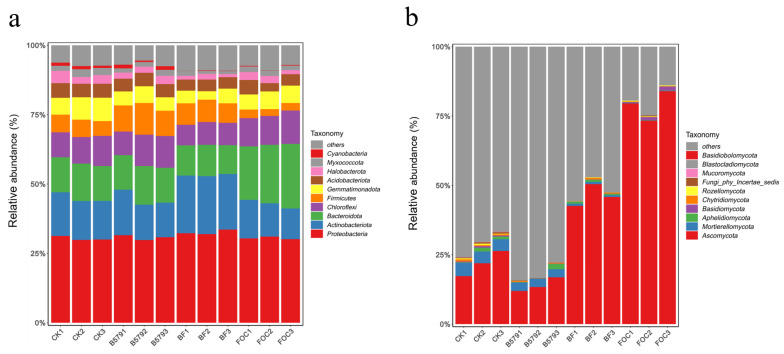
The relative abundance of the dominant phyla in the cucumber rhizosphere soil samples: (**a**) bacteria; (**b**) fungi. CK (cucumbers irrigated with distilled water (control)); B579 (cucumbers irrigated with B579 suspension (2 × 10^8^ CFU/mL)); B579 + FOC (BF) (cucumbers irrigated with B579 suspension for 3 days and subsequently with FOC suspension (1 × 10^8^ conidia/mL)); FOC (cucumbers irrigated with FOC suspension).

An analysis of the bacterial sequencing results across all samples revealed 10 bacterial phyla and 10 fungal phyla, while *Firmicutes* (2.72–11.40%) was not the richest bacterial phylum. Among the classified bacterial phyla, *Proteobacteria* occupied the highest proportion (29.81–33.58%), followed by *Actinobacteria* (11.08–20.92%), *Bacteroidetes* (10.37–23.29%), *Chloroflexi* (7.42–12.02%), and *Firmicutes* (2.45–11.40%), in each sample (Figure 6a). *Firmicutes* exhibited a significantly higher relative abundance in the bacterial phyla in the B579 treatment compared to that under the control treatment. Additionally, the pretreatment with B579 (B579 + FOC) significantly increased the relative abundance of *Proteobacteria*, *Actinobacteriota*, and *Firmicutes* (*p* < 0.05) while reducing that of *Bacteroidota* and *Chloroflexi* (*p* < 0.05) compared to these values under the FOC treatment (Table S1).

The analysis of the fungal sequencing results showed that *Ascomycota* (11.92–83.93%) accounted for the highest proportion of the total fungi, succeeded by *Mortierellomycota* (0.11–4.73%) and *Basidiomycota* (0.04–1.47%), in each sample (Figure 6b). Nevertheless, the irrigation with FOC altered the fungal composition in the cucumber rhizosphere soil. Following inoculation with the pathogen (B579 + FOC and FOC), a notable decline in the relative abundance of unclassified fungi was detected, whereas *Ascomycota* showed the opposite trend.

Compared with the other treatments, *Ascomycota* exhibited the highest relative abundance in the FOC-treated soil (78.94%) (*p* < 0.05). Furthermore, B579 pretreatment markedly reduced the relative abundance of *Ascomycota* relative to that under the FOC treatment (Table S2).

**Figure 7:**
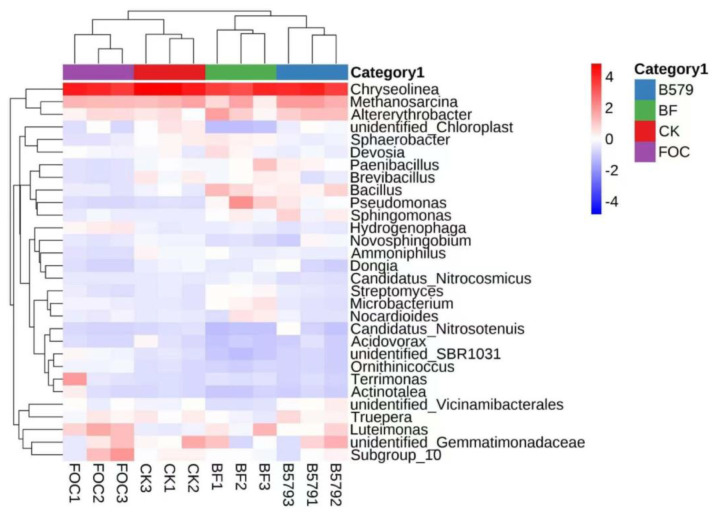
A heatmap of the top 30 classified bacterial genera for the samples in different treatments. CK (cucumbers irrigated with distilled water (control)); B579 (cucumbers irrigated with B579 suspension (2 × 10^8^ CFU/mL)); B579 + FOC (BF) (cucumbers irrigated with B579 suspension for 3 days and subsequently with FOC suspension (1 × 10^8^ conidia/mL)); FOC (cucumbers irrigated with FOC suspension). Data were normalized between groups, with red representing the high relative abundance of a species and blue representing low abundance of that species.

At the genus level, we detected a total of 708 bacterial genera and 243 fungal genera in all samples. Based on the ASVs, a cluster analysis for the top 30 abundant bacterial genera and the top 15 fungal genera was applied, and a hierarchical heatmap was generated. Among the top 30 categorized bacterial genera, several previously reported beneficial genera were identified. We selected six representative bacterial genera: *Bacillus*, *Paenibacillus*, *Sphingomonas*, *Pseudomonas*, *Microbacterium*, and *Streptomyces* (Figure 7). Compared to the control treatment, B579 treatment significantly increased the relative abundance of *Bacillus*, *Sphingomonas*, and *Pseudomonas* (*p* < 0.05). Moreover, all six bacterial genera were significantly more abundant in the BF treatment than in the FOC treatment (*p* < 0.05) (Table 5).

**Figure 8:**
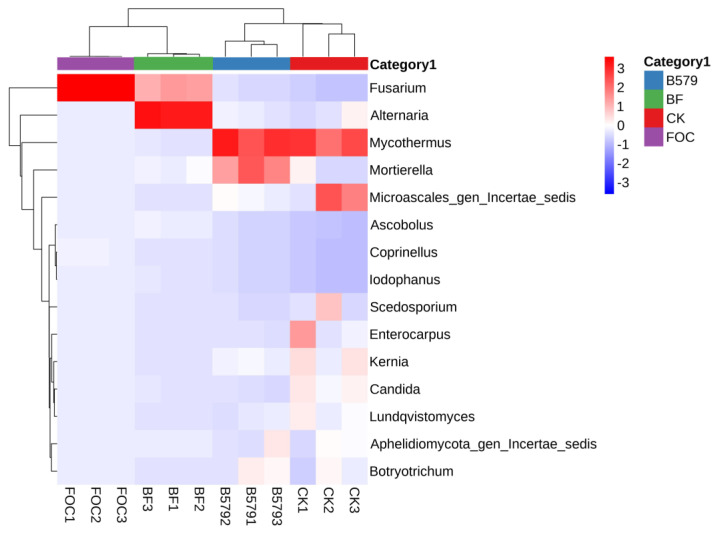
A heatmap of the top 15 classified fungal genera for the samples in different treatments. CK (cucumbers irrigated with distilled water (control)); B579 (cucumbers irrigated with B579 suspension (2 × 10^8^ CFU/mL)); B579 + FOC (BF) (cucumbers irrigated with B579 suspension for 3 days and subsequently with FOC suspension (1 × 10^8^ conidia/mL)); FOC (cucumbers irrigated with FOC suspension).

Concerning fungi, exogenous microorganisms altered the composition of the original microbial community, and *Fusarium* was the dominant fungal species in the BF-treated (48.34%) and FOC-treated soil (75.44%) (Figure 8). Compared to the FOC treatment, the BF (B579 + FOC) treatment significantly decreased the relative abundance of *Fusarium* (*p* < 0.05). *Mortierella* in the fungal genera exhibited a significantly higher relative abundance in the BF treatment compared to that under the FOC treatment (*p* < 0.05). In addition, among the top 30 fungal genera, *Trichoderma* showed the highest content in the BF treatment compared with that in the other treatments (*p* < 0.05) (Table 6).

### 3.7. The Correlation Between the Soil’s Physicochemical Properties and Abundant Genera

**Figure 9:**
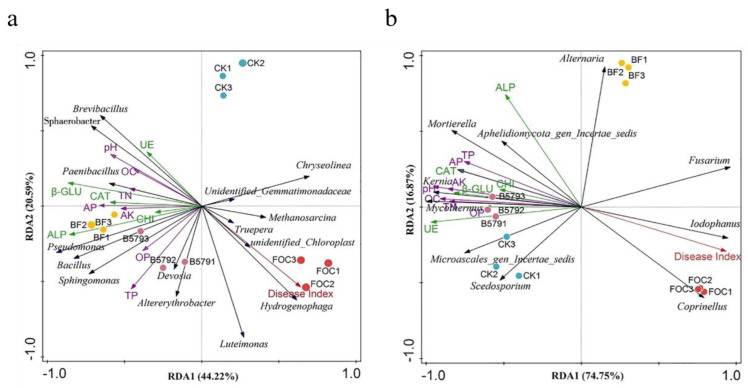
The redundancy analysis (RDA) based on the major genera and the soil’s physicochemical properties in the rhizosphere soil of cucumber. (**a**) Bacteria; (**b**) fungi. CK (cucumbers irrigated with distilled water (control)); B579 (cucumbers irrigated with B579 suspension (2 × 10^8^ CFU/mL)); B579 + FOC (BF) (cucumbers irrigated with B579 suspension for 3 days and subsequently with FOC suspension (1 × 10^8^ conidia/mL)); FOC (cucumbers irrigated with FOC suspension). TP, total phosphorus; TN, total nitrogen; OC, organic carbon; AK, available potassium; AP, available phosphorus; OP, organic phosphorus; UE, urease; ALP, alkaline phosphatase; CAT, catalase; CHI, chitinase; β-GLU, β-glucosidase.

The correlation between 12 of the soil’s physicochemical properties and bacterial/fungal community diversity was analyzed using an RDA (Figure 9). The RDA model explained 64.81% of the total bacterial variation (Figure 9a), with RDA1 and RDA2 accounting for 44.22% and 20.59% of the variation, respectively. The bacterial communities in the BF treatment showed strong correlations with elevated soil pH and TN, AP, TP, OC, AK contents, while the bacterial communities under the FOC treatment highly correlated with the disease index. All of the soil enzyme activities measured had a significant influence on the bacterial communities under both the B579 and B579 + FOC conditions. Additionally, the genera *Bacillus*, *Paenibacillus*, *Sphingomonas*, and *Pseudomonas* were positively associated with enzyme activities, pH level, and the contents of TN, AP, TP, OC, and AK. For the fungal genera (Figure 9b), the first two RDA axes explained 74.75% and 16.87% of the variation, respectively. The abundance of *Mortierella* was positively correlated with all soil enzyme activities, and the abundance of *Fusarium* was strongly related to the disease index.

## 4. Discussion

The main obstacle in cucumber production is the aggravation of soil-borne diseases, of which cucumber wilt is the most serious one [43]. Studies have indicated that the application of *B. subtilis* effectively controls fungal diseases through producing broad-range inhibitory effects, particularly for fusarium wilt [44,45,46]. However, their colonization ability, as a prerequisite, determines whether they can perform their special functions [47]. *B. subtilis* B579 was confirmed as able to effectively colonize cucumber rhizosphere soil through combining antibiotic plate recovery and Laser Scanning Confocal Microscope (LSCM) testing [48]. In this study, the cucumber seedlings irrigated with the B579 suspension exhibited enhanced plant heights, weights, and stem diameters in comparison to these values in those irrigated with distilled water (Figure 2). Additionally, pretreatment with B579 (BF) significantly reduced the disease index to 13.89% compared to that under the FOC treatment (*p* < 0.05) (Figure 1). These findings are in compliance with previous studies where *Bacillus subtilis* was utilized to facilitate plant growth and suppress fungal plant diseases [49,50].

Soil enzymes and physicochemical properties are key components of the soil’s ecological processes and play important roles in soil metabolism. In this study, the urease activity in the soil samples under the BF treatment was significantly higher than that under the FOC treatment (*p* < 0.05), and the TN content showed a similar trend. Consistent with the results reported by Hao et al. [51] in an analysis of the soil properties in paddy, urease activity was positively correlated with the TN content (Figure 9). In the BF treatment, both the soil’s alkaline phosphatase (ALP) activity and AP content were significantly greater than those in the FOC treatment. These results align with previous reports indicating that the introduction of *B. subtilis* was consistently associated with increased ALP activity and plant-available phosphate content [52]. Furthermore, the BF-treated soil exhibited significantly elevated pH levels alongside increased contents of TP, OC, and AK, as well as enhanced CAT and β-GLU activities, relative to those in the FOC-treated soil. Interestingly, no significant differences in physicochemical properties were observed between the disinfected and control soil. However, the application of B579 obviously improved the soil’s enzyme activities (UR, ALP, CAT, and β-GLU) and physicochemical properties (pH, TN, OC, AK, AP, and OP) in the same period. Bai et al. [53] found that irrigation of *B. subtilis* A-5 changed the microbial community structure in the rhizosphere soil of Chinese cabbage, increasing the soil’s fertility and further facilitating growth in Chinese cabbage. Our findings are consistent with previous studies showing that the use of *Bacillus* inoculants can alter the soil’s microbial community structure and improve the soil’s enzyme activity and nutrient availability [54,55].

The rhizosphere microbiota often rapidly adapt to environmental changes, contributing significantly to disease suppression and health maintenance in plants [56]. First, when plant roots are attacked by pathogens, plants can selectively alter the rhizosphere’s microbial populations for self-protection [57]. So, B579 inoculation may produce a selective effect on the rhizosphere’s microbial communities, which, in turn, alters various aspects of the soil environment. Secondly, FOC inoculation can lead to the colonization of the rhizosphere by FOC populations, which also may affect the composition of the microbial community [58]. Under the support of these two theories, we focused on the effects of B579 application on the rhizosphere microbial community for cucumber under *Fusarium oxysporum* invasion and explored the relationship between microbial community regulation and Fusarium wilt control.

No statistically significant differences were observed in the chao1 value or Shannon and Simpson indices for bacteria and fungi between the non-inoculated (CK) soil and the B579-treated soil, implying that the use of B579 alone did not pronouncedly influence the diversity of the cucumber rhizosphere microbiome (Table 4). Similarly, Tu et al. [27] found that the single inoculation of *B. subtilis* Y37 did not significantly modify the microbial diversity of the lily rhizosphere. Unlike chemical fungicides which reduce the soil’s microbial diversity, biocontrol agents demonstrate a superior capacity to maintain microbiome stability [59,60]. Furthermore, in comparison to the FOC treatment, the BF (B579 + FOC) treatment significantly improved the soil’s bacterial and fungal community diversity. These results are consistent with previous studies demonstrating that increased microbial diversity of the rhizosphere strengthens cucumbers’ resistance to Fusarium wilt [61,62].

The microbial community analysis revealed *Proteobacteria*, *Actinobacteria*, *Bacteroidota*, and *Chloroflexi* as the four most dominant phyla in all of the rhizosphere soil samples (Figure 6a). This is consistent with another study that showed that *Proteobacteria*, *Actinobacteria*, *Chloroflexi,* and *Bacteroidota* were the dominant phyla in the rhizosphere soil for cucumbers, with *Proteobacteria* accounting for the highest proportion (28–35%) [63]. The B579-treated soil exhibited a high relative abundance of the *Bacillus*, *Paenibacillu*s, *Sphingomonas*, *Pseudomonas*, *Microbacterium*, and *Streptomyces* genera. Compared to the FOC treatment, their relative abundance was prominently increased in the B579 + FOC treatment (*p* < 0.05). The genera *Bacillus*, *Paenibacillus,* and *Pseudomonas* are well-known BCAs in agricultural soil and have frequently been reported to be responsible for suppressing soil-borne pathogens and promoting plant growth by secreting antifungal metabolites, competing for niches and nutrients, producing phytohormones, and using root exudates [64,65,66,67]. Especially, the genera *Bacillus* and *Paenibacillu*s are members of the *Firmicutes* phylum, which encompasses a multitude of potential BCAs. It has been reported that the ratio of *Firmicutes* tends to be high in suppressive soil [68,69]. The genera *Microbacterium* and *Streptomyces* fall under the *Actinobacteria* phylum, which constitutes a significant portion of microbial rhizosphere communities. Notably, non-pathogenic *Actinobacteria*, including *Microbacterium* and *Streptomyces*, are known to trigger systemic acquired resistance (SAR) to pathogens in plants [70,71]. In addition, the relative abundance of *Sphingomonas* in the BF treatment was significantly higher than that in the FOC treatment (*p* < 0.05). Studies have shown that *Sphingomonas* can secrete endochitinase to degrade β-1, 4-glycosidic bonds in the cell walls of pathogenic fungi and synthesize high-affinity siderophores such as hydroxamic acid to deprive pathogens of iron supply [72]. Combined with previous studies, our findings indicate that B579 plays a crucial role in modulating the soil’s microbial community, particularly by enhancing the abundance of *Bacillus*, *Paenibacillu*s, *Sphingomonas*, *Pseudomonas*, *Microbacterium*, and *Streptomyces*, which are involved in the suppression of plant diseases. However, whether this mechanism is direct or indirect remains unclear and requires further investigation.

Additionally, the suppressive effect of B579 on Fusarium wilt was also achieved via the modulation of the fungal community’s composition. *Ascomycota* emerged as the most dominant phylum in all samples, followed by *Mortierellomycota* and *Basidiomycota* (Figure 6b), consistent with previous reports that *Ascomycota* was dominant in cucumber rhizosphere soil [3]. *Ascomycota* contains many fungal pathogens [73], and the relative abundance of *Ascomycota* in the soil samples under the B579 + FOC treatment was significantly lower than that under the FOC treatment (*p* < 0.05). As for the fungal genera, compared to the FOC treatment, B579 pretreatment (B579 + FOC) markedly decreased the abundance of the *Fusarium* species responsible for Fusarium wilt (*p* < 0.05). Additionally, the genera *Mortierella* and *Trichoderma* were also more abundant in the B579 + FOC treatment than in the FOC treatment (*p* < 0.05). *Trichoderma* is an important source of BCAs due to their biological control properties, such as mycoparasitism, the secretion of antagonistic compounds, and induction of plants’ defense systems [74]. The lytic enzymes secreted by *Mortierella* can degrade structural polysaccharides such as chitin, a major component of fungal cell walls [75]. Studies have also confirmed that *Mortierella* promotes plant growth and provides higher resistance to soil-borne pathogens [76]. These findings demonstrate that the application of B579 shapes a microbial community structure in the rhizosphere soil that is unfavorable for pathogen growth.

The RDA revealed correlations between the soil environmental variables and major bacterial and fungal genera. The TP content was higher in the B579 + FOC-treated soil than that in the FOC-treated soil. Furthermore, the disease index exhibited a positive correlation with FOC treatment (Figure 9b). These findings align with those of Shi et al. [62], who reported a strong association between a high phosphorus content in the rhizosphere soil and a lower disease index for Rhizoctonia triticum. In addition, soil pH was negatively related to the disease index, and the soil pH in the BF treatment was significantly higher than that in the FOC treatment. Many studies have shown that a high pH is often present in suppressive soil [61]. However, other studies have found that the disease severity score declines as the pH decreases, with no correlation between the pH and the severity of plant disease [77]. Hence, the relationship between soil pH and the occurrence of plant disease may vary depending on the plant species and soil type studied. CAT and β-GLU activities were high in the B579-treated soil, showing a negative relationship with the disease index. CAT can alleviate the oxidative damage to plants caused by reactive oxygen species (ROS) under pathogen stress by decomposing hydrogen peroxide (H_2_O_2_) [78]. Studies have reported that β-glucosidase hydrolyzes the glycosidic bonds in plant cell walls, releasing defense signaling molecules (e.g., salicylic acid precursors, flavonoids), which activate systemic acquired resistance (SAR) [79]. Notably, the glucose generated through the β-glucosidase-mediated decomposition of organic matter may serve as a carbon source, promoting the growth of beneficial microorganisms, such as *Pseudomonas* and *Trichoderm* [80]. Moreover, the *Bacillus*, *Paenibacillus*, *Sphingomonas*, *Pseudomonas*, and *Mortierella* genera were positively correlated with pH level; TP, TN, OC, AK, and AP contents; and enzyme activities (UR, ALP, CAT, and β-GLU), whereas *Fusarium* was negatively associated with these soil properties, indicating that the application of B579 did not favor *Fusarium* growth. Given that cucumber is primarily cultivated under field conditions, further field trials are necessary to evaluate its practical control efficacy. Additionally, a plant rhizosphere’s microbial community is susceptible to influence by variations in the soil type and environmental conditions. Therefore, further studies are needed to investigate the effects of this specific strain (B579) on the soil’s microbial community under different soil types and climatic conditions.

In the present study, pretreatment with strain B579 significantly enhanced the soil’s physicochemical properties (pH, TP, TN, OC, AK, AP) and enzyme activities (UR, ALP, CAT, and β-GLU) compared with those under the FOC treatment. The RDA indicated that these soil properties were positively associated with plant disease resistance. The application of B579 may systematically modulate plant–soil interactions by increasing the diversity and relative abundance of beneficial rhizosphere microorganisms, including the genera *Bacillus*, *Paenibacillus*, *Sphingomonas*, *Pseudomonas*, *Microbacterium*, *Mortierella*, and *Trichoderma*, while concomitantly reducing the severity of Fusarium wilt and promoting cucumber growth. Moreover, the relative abundance of *Fusarium* was significantly lower in the BF-treated soil relative to that in the FOC-treated soil. The rhizosphere soil’s properties are collectively modulated by microbial communities and root exudates. Nevertheless, the interplay within the plant–soil–microbe triad exhibits profound complexity, representing a priority research direction for future studies. To sum up, B579 application can alter the soil’s enzyme activities, physicochemical properties, and microbial community composition, creating an appropriate soil environment for disease suppression.

## 5. Conclusions

In the pot experiment, B579 achieved a disease control efficiency of 73.86% against Fusarium wilt in cucumbers. B579 pretreatment significantly enhanced both the bacterial and fungal diversity and enriched beneficial taxa such as the *Bacillus*, *Paenibacillu*s, *Sphingomonas*, *Pseudomonas*, *Microbacterium*, *Mortierella,* and *Trichoderma* genera compared to the FOC treatment. Additionally, the application of the B579 strain affected the soil’s physicochemical properties (pH, OC, TN, TP, AK, and AP) and enzyme activities, especially those of urease and alkaline phosphatase. Interestingly, the RDA results showed that the use of *B. subtilis* B579 is not conducive to the growth of the pathogen, thus reducing the severity of Fusarium wilt in cucumbers further. This study provides novel insights for understanding the disease suppression mechanisms of *Bacillus subtilis* B579.

## Figures and Tables

**Table 1:** The physicochemical properties of cucumber rhizosphere soil in different treatments.

Treatment	pH	TP (μg/g)	TN (g/kg)	OC (g/kg)	AK (mg/kg)	AP (mg/kg)	OP (μg/g)
CK	7.47 ± 0.05 ^b^	1147.83 ± 131.48 ^b^	1.34 ± 0.13 ^b^	32.60 ± 0.16 ^b^	79.58 ± 6.57 ^b^	38.61 ± 2.82 ^b^	529.91 ± 28.05 ^b^
B579	7.70 ± 0.07 ^a^	1340.28 ± 58.17 ^b^	1.59 ± 0.02 ^a^	34.54 ± 1.30 ^a^	112.17 ± 6.21 ^a^	57.28 ± 5.92 ^a^	652.92 ± 6.88 ^a^
B579 + FOC	7.19 ± 0.04 ^c^	1140.12 ± 66.22 ^a^	1.32 ± 0.07 ^b^	30.71 ± 0.84 ^c^	71.69 ± 4.16 ^b^	38.35 ± 1.67 ^b^	528.48 ± 7.25 ^b^
FOC	6.80 ± 0.06 ^d^	968.53 ± 32.81 ^c^	1.10 ± 0.05 ^c^	28.81 ± 0.80 ^d^	58.07 ± 5.60 ^c^	26.34 ± 1.81 ^c^	522.99 ± 9.11 ^b^

Note: Values represent the mean ± SE (n = 3). CK (cucumbers irrigated with distilled water (control)); B579 (cucumbers irrigated with the B579 suspension (2 × 10^8^ CFU/mL)); B579 + FOC (BF) (cucumbers irrigated with the B579 suspension for 3 days and subsequently with the FOC suspension (1 × 10^8^ conidia/mL)); FOC (cucumbers, irrigated with the FOC suspension). TP, total phosphorus; TN, total nitrogen; OC, organic carbon; AK, available potassium; AP, available phosphorus; OP, organic phosphorus. Values marked with different letters within a column are significantly different according to Tukey’s test (*p* < 0.05).

**Table 2:** The enzyme activities of cucumber rhizosphere soil under different treatments.

Treatment	Urease (mg/kg·h)	Alkaline Phosphatase (mg/g·h)	Catalase (mL/g·h)	Chitinase (μg/g·h)	β-Glucosidase (μg/g·h)
CK	27.44 ± 2.21 ^b^	1.08 ± 0.05 ^b^	3.79 ± 0.17 ^b^	2.54 ± 0.34 ^a^	157.64 ± 11.94 ^b^
B579	32.14 ± 1.53 ^a^	1.49 ± 0.13 ^a^	4.57 ± 0.16 ^a^	2.88 ± 0.16 ^a^	188.42 ± 12.02 ^a^
B579 + FOC	23.73 ± 1.86 ^c^	1.45 ± 0.11 ^a^	3.74 ± 0.33 ^b^	2.51 ± 0.25 ^a^	145.82 ± 5.26 ^b^
FOC	13.39 ± 0.63 ^d^	0.88 ± 0.06 ^c^	3.15 ± 0.18 ^c^	2.37 ± 0.36 ^a^	124.64 ± 3.24 ^c^

Note: Values represent the mean ± SE (n = 3). CK (cucumbers irrigated with distilled water (control)); B579, (cucumbers irrigated with the B579 suspension (2 × 10^8^ CFU/mL)); B579 + FOC (BF) (cucumbers irrigated with the B579 suspension for 3 days and subsequently with the FOC suspension (1 × 10^8^ conidia/mL)); FOC (cucumbers irrigated with FOC suspension). Values marked with different letters within a column are significantly different according to Tukey’s test (*p* < 0.05).

**Table 3:** Number of sequences retained after splicing, filtering, and chimera removal across different treatments.

Sample	Retained Sequences	
	Bacterial 16S rRNA Sequences	Fungal ITS Sequences
CK1	77,149	105,995
CK2	71,265	98,215
CK3	75,819	94,054
B5791	82,872	97,147
B5792	79,211	104,539
B5793	75,083	94,954
B579 + FOC1 (BF1)	119,309	100,749
B579 + FOC2 (BF2)	85,392	99,151
B579 + FOC3 (BF3)	82,269	105,446
FOC1	69,980	98,040
FOC2	68,742	101,698
FOC3	72,079	98,244
Mean	79,931	99,853
Total	959,170	1,198,232

**Table 4:** The microbial diversity index of the rhizosphere soil in different treatments.

Treament	Community Characteristics					
	Bacterial Community			Fungal Community		
**CK**	chao1	Shannon	Simpson	chao1	Shannon	Simipson
	2157.04 ± 299.12 ^ab^	9.39 ± 0.34 ^ab^	0.99 ± 0.00 ^ab^	360.76 ± 73.76 ^a^	4.15 ± 0.69 ^a^	0.86 ± 0.05 ^a^
**B579**	2497.08 ± 111.19 ^a^	9.73 ± 0.21 ^a^	0.99 ± 0.00 ^a^	215.66 ± 20.32 ^ab^	2.70 ± 0.56 ^ab^	0.74 ± 0.08 ^ab^
**B579 + FOC**	2102.28 ± 325.81 ^ab^	9.28 ± 0.43 ^ab^	0.99 ± 0.00 ^ab^	247.02 ± 24.47 ^ab^	3.33 ± 0.80 ^ab^	0.79 ± 0.08 ^ab^
**FOC**	1719.89 ± 157.17 ^b^	8.97 ± 0.27 ^c^	0.99 ± 0.00 ^c^	143.40 ± 27.32 ^b^	1.70 ± 0.34 ^c^	0.42 ± 0.08 ^c^

Note: Values represent the mean ± SE (n = 3). CK (cucumbers irrigated with distilled water (control)); B579 (cucumbers irrigated with B579 suspension (2 × 10^8^ CFU/mL)); B579 + FOC (BF) (cucumbers irrigated with B579 suspension for 3 days and subsequently with FOC suspension (1 × 10^8^ conidia/mL)); FOC (cucumbers irrigated with FOC suspension); Values marked with different letters within a column are significantly different according to Tukey’s test (*p* < 0.05).

**Table 5:** Analysis of significant differences in potentially beneficial bacterial genera (%) among different treatments.

Taxonomy	CK	B579	BF	FOC
Bacillus	0.56 ± 0.14 ^b^	1.38 ± 0.18 ^a^	1.36 ± 0.37 ^a^	0.48 ± 0.10 ^b^
Paenibacillus	0.70 ± 0.03 ^ab^	1.16 ± 0.30 ^a^	1.08 ± 0.47 ^a^	0.30 ± 0.10 ^b^
Sphingomonas	0.42 ± 0.06 ^b^	1.23 ± 0.51 ^a^	0.85 ± 0.20 ^a^	0.52 ± 0.19 ^b^
Pseudomonas	0.20 ± 0.01 ^b^	1.01 ± 0.30 ^a^	1.53 ± 0.43 ^a^	0.16 ± 0.04 ^b^
Microbacterium	0.35 ± 0.06 ^b^	0.45 ± 0.08 ^b^	1.03 ± 0.15 ^a^	0.59 ± 0.04 ^b^
Streptomyces	0.34 ± 0.03 ^b^	0.39 ± 0.06 ^b^	0.54 ± 0.22 ^a^	0.36 ± 0.08 ^b^

Note: Values represent the mean ± SE (n = 3). CK (cucumbers irrigated with distilled water (control)); B579 (cucumbers irrigated with B579 suspension (2 × 10^8^ CFU/mL)); B579 + FOC (BF) (cucumbers irrigated with B579 suspension for 3 days and subsequently with FOC suspension (1 × 10^8^ conidia/mL)); FOC (cucumbers irrigated with FOC suspension). Values marked with different letters within a row are significantly different according to Tukey’s test (*p* < 0.05).

**Table 6:** Analysis of significant differences in fungal genera associated with plant diseases (%) among different treatments.

Taxonomy	CK	B579	BF	FOC
Fusarium	0.11 ± 0.03 ^c^	0.08 ± 0.02 ^c^	48.34 ± 7.98 ^b^	75.44 ± 5.74 ^a^
Mortierella	0.53 ± 0.10 ^bc^	3.21 ± 0.35 ^a^	0.77 ± 0.27 ^b^	0.01 ± 0.00 ^c^
Trichoderma	0.05 ± 0.00 ^b^	0.06 ± 0.01 ^b^	0.10 ± 0.02 ^a^	0.05 ± 0.01 ^b^

Note: Values represent the mean ± SE (n = 3). CK (cucumbers irrigated with distilled water (control)); B579 (cucumbers irrigated with B579 suspension (2 × 10^8^ CFU/mL)); B579 + FOC (BF) (cucumbers irrigated with B579 suspension for 3 days and subsequently with FOC suspension (1 × 10^8^ conidia/mL)); FOC (cucumbers irrigated with FOC suspension). Values marked with different letters within a row are significantly different according to Tukey’s test (*p* < 0.05).

## Data Availability

The original contributions presented in this study are included in the article/Supplementary Material. Further inquiries can be directed to the corresponding author.

## Supplementary Materials

The following supporting information can be downloaded at: https://www.mdpi.com/article/10.3390/microorganisms13061382/s1, Table S1: Relative abundance (%) of main classified bacterial phylum in soil samples; Table S2: Relative abundance (%) of main classified fungal phylum in soil samples; Table S3: Relative abundance (%) of the top 40 classified bacterial genera in soil samples; Table S4: Relative abundance (%) of the top 20 classified fungal genera in soil samples.
