# Supplementary material for: Bacillus subtilis B579 Controls Cucumber Fusarium Wilt by Improving Rhizosphere Microbial Community
Source: Microorganisms. 2025 Jun 13;13(6):1382. doi: 10.3390/microorganisms13061382 (PMC12196314; doi:10.3390/microorganisms13061382)
Supplement: Supplementary file 1 [file microorganisms-13-01382-s001.zip › Supplementary Material.pdf]

Bacillus subtilis B579 controls cucumber Fusarium wilt by improving the rhizosphere microbial community

Zongqiang Fan [1], Jinghan Feng [1], Lixue Zheng [1,2], Yanru Chen [1], Minglei Wang [1], Xiangqian Peng [1], Shuo Wang [1,3], and Fang Chen [1],*

[1] School of Pharmaceutical Sciences and Food Engineering, Liaocheng University, Liaocheng, 252000, China

[2] State Key Laboratory of Macromolecular Drugs and Large-scale Preparation, Liaocheng University, Liaocheng, 252000, China

[3] Shandong Key Laboratory of Applied Technology for Protein and Peptide Drugs, Liaocheng University, Liaocheng, 252000, China

*Corresponding author: Fang Chen; E-mail: chenfang20045@126.com

Supplementary Table S1 Relative abundance（%）of main classified bacterial phylum in soil samples

Taxonomy	CK	B579	BF	FOC
Proteobacteria	30.37±0.81a	30.73±0.88ab	32.59±0.88b	30.51±0.49a
Actinobacteriota	14.57±1.01a	13.86±2.19a	20.59±0.47b	12.34±1.46a
Bacteroidota	12.89±0.52a	12.97±0.82a	10.85±0.46a	21.20±2.03b
Chloroflexi	9.79±0.99ab	10.43±1.66ab	7.88±0.41a	10.85±1.02b
Firmicutes	6.00±0.57b	9.96±1.26c	7.62±0.55b	2.77±0.34a
Gemmatimonadota	7.53±1.27b	5.33±0.63ab	4.28±1.16a	6.02±0.53ab
Acidobacteriota	5.05±0.22a	4.72±0.13a	4.13±0.09a	4.11±1.10a
Halobacterota	3.32±1.00a	2.47±0.45a	1.50±0.48a	2.30±0.76a
Myxococcota	2.41±0.50b	1.77±0.32ab	1.30±0.388a	1.88±0.19ab
Cyanobacteria	1.03±0.19b	1.05±0.47b	0.12±0.06a	0.17±0.08a

Note: Values represent the mean ± SE (n = 3); Values marked with different letters within a row are significantly different according to Tukey's test ($p < 0.05$).

Supplementary Table S2 Relative abundance（%）of main classified bacterial phylum in soil samples

Taxonomy	CK	B579	BF	FOC
Ascomycota	21.86±4.52a	14.04±2.54a	46.28±3.94b	78.94.±5.32c
Mortierellomycota	4.39±0.30d	2.99±0.05c	0.76±0.03b	0.13±0.01a
Basidiomycota	0.46±0.11ab	0.08±0.03a	0.10±0.03ab	0.93±0.63b
Aphelidiomycota	0.83±0.59b	0.71±0.38b	0.64±0.15b	0.08±0.06a
Rozellomycota	0.37±0.16b	0.03±0.02a	0.09±0.04ab	0.26±0.02ab
Fungi_phy_Incertae_sedis	0.47±0.14b	0.11±0.04a	0.04±0.02a	0.10±0.17a
Chytridiomycota	0.52±0.21a	0.20±0.15a	0.33±0.32a	0.14±0.11a
Mucoromycota	0.07±0.03b	0.02±0.03a	0.01±0.00a	0.00±0.00a
Blastocladiomycota	0.01±0.06a	0.01±0.00a	0.01±0.01a	0.01±0.00a
Basidiobolomycota	0.01±0.00a	0.01±0.00a	0.00±0.00a	0.00±0.00a

Note: Values represent the mean ± SE (n = 3); Values marked with different letters within a row are significantly different according to Tukey's test ($p < 0.05$).

Supplementary Table S3 Relative abundance（%）of the top 40 classified bacterial genera in soil samples

Taxonomy	CK	B579	BF	FOC
Chryseolinea	5.48±0.07a	4.83±1.02ab	3.36±0.37a	5.52±0.97a
Methanosarcina	2.20±0.11a	2.71±0.53a	1.44±0.47b	2.44±0.29a
Altererythrobacter	1.14±0.36a	2.07±0.32a	1.46±0.55a	1.50±0.18a
unidentified_Gemmatimonadaceae	1.36±0.77a	1.51±0.98a	0.92±0.60b	1.28±0.95a
Luteimonas	0.44±0.12c	1.20±0.32b	1.23±0.53b	2.25±0.21a
Truepera	1.01±0.31a	1.29±0.29a	0.66±0.13b	1.05±0.25a
Bacillus	0.56±0.14b	1.38±0.18a	1.36±0.37a	0.48±0.10b
Paenibacillus	0.70±0.03ab	1.16±0.30a	1.08±0.47a	0.30±0.10b
Sphingomonas	0.42±0.06b	1.23±0.51a	0.85±0.20a	0.52±0.19ab
Devosia	0.61±0.26a	0.70±0.09a	0.95±0.30a	0.76±0.13a
Sphaerobacter	0.92±0.11a	0.71±0.07ab	1.02±0.12a	0.40±0.19b
unidentified_Vicinamibacterales	0.63±0.18ab	1.06±0.14a	0.41±0.03b	0.84±0.27ab
Pseudomonas	0.20±0.01b	1.01±0.30a	1.53±0.43a	0.16±0.04b
Hydrogenophaga	0.47±0.14b	0.54±0.09b	0.61±0.07b	1.25±0.16a
Brevibacillus	1.01±0.35a	0.69±0.46a	0.90±0.19a	0.27±0.04a
Ammoniphilus	0.75±0.27a	0.67±0.06a	0.68±0.14a	0.25±0.05b
Microbacterium	0.35±0.06b	0.45±0.08b	1.03±0.15a	0.59±0.04b
Dongia	0.56±0.08a	0.42±0.54a	0.61±0.07a	0.05±0.00a
Ornithinicoccus	0.17±0.05b	0.17±0.04b	0.29±0.02ab	0.68±0.06a
Candidatus_Nitrocosmicus	0.39±0.01b	0.38±0.01b	0.64±0.10a	0.41±0.01b
Terrimonas	0.14±0.05a	0.25±0.04a	0.45±0.01a	1.39±1.74a
unidentified_Chloroplast	1.07±0.26a	0.84±0.23a	0.03±0.00b	0.37±0.47ab
unidentified_SBR1031	0.27±0.06a	0.17±0.05a	0.08±0.01a	0.85±0.24b
Actinotalea	0.12±0.00a	0.13±0.01a	0.17±0.06a	0.57±0.07a
Nocardioides	0.40±0.01a	0.55±0.20a	0.91±0.43a	0.53±0.12a
Novosphingobium	0.56±0.09a	0.68±0.51a	0.40±0.08a	0.40±0.08a
Subgroup_10	0.92±0.12a	0.80±0.38a	0.78±0.05a	1.84±1.36a
Candidatus_Nitrosotenuis	0.41±0.49a	0.21±0.19a	0.07±0.07a	0.00±0.01a
Acidovorax	0.25±0.05a	0.37±0.12a	0.12±0.10a	0.14±0.00a
Ramlibacter	0.05±0.00b	0.13±0.04b	0.17±0.02b	0.42±0.13a
Arenimonas	0.31±0.29a	0.59±0.32a	0.52±0.34a	0.20±0.08a
Dokdonella	0.41±0.20a	0.61±0.39a	0.53±0.25a	0.70±0.11a
Gemmatimonas	0.56±0.01b	0.80±0.12a	0.63±0.05b	0.35±0.06c
Pedomicrobium	0.76±0.01ab	0.80±0.15ab	0.88±0.04a	0.64±0.18b
Streptomyces	0.34±0.03b	0.39±0.06b	0.54±0.22a	0.36±0.08b
Pelagibius	0.32±0.19a	0.41±0.16a	0.42±0.38a	0.33±0.28a
Candidatus_Chloroploca	0.26±0.16a	0.43±0.33b	0.39±0.31b	0.35±0.18a
Nonomuraea	0.39±0.08a	0.34±0.14a	0.53±0.26a	0.39±0.13a
Thermobispora	0.44±0.09a	0.29±0.06a	0.45±0.27a	0.20±0.11a
MND1	0.56±0.17a	0.31±0.03a	0.22±0.01b	0.15±0.12b

Note: Values represent the mean ± SE (n = 3); Values marked with different letters within a row are significantly different according to Tukey's test (p < 0.05).

Supplementary Table S4 Relative abundance（%）of the top 20 classified fungal genera in soil samples

Taxonomy	CK	B579	BF	FOC
Fusarium	0.11±0.03c	0.08±0.02c	48.34±7.98b	75.44±5.74a
Alternaria	0.70±0.50b	0.41±0.09b	11.17±1.93a	0.29±0.04b
Mortierella	0.53±0.10bc	3.21±0.35a	0.77±0.27b	0.01±0.00c
Mycothermus	3.32±0.95a	4.85±1.59a	0.18±0.03b	0.01±0.00b
Microascales_gen_Incertae_sedis	2.57±2.18a	0.64±0.17a	0.14±0.03a	0.15±0.03a
Coprinellus	0.05±0.01b	0.02±0.01b	0.03±0.00b	1.19±0.27a
Scedosporium	1.05±1.18a	0.07±0.01a	0.03±0.01a	0.06±0.01a
Iodophanus	0.05±0.01b	0.01±0.00b	0.15±0.07b	0.75±0.09a
Kernia	1.03±0.37a	0.52±0.04b	0.15±0.02bc	0.02±0.01c
Aphelidiomycota_gen_Incertae_sedis	0.82±0.59a	0.58±0.79a	0.51±0.04a	0.07±0.01a
Enterocarpus	0.98±0.35a	0.20±0.05b	0.06±0.01b	0.10±0.03b
Botryotrichum	0.75±0.68a	0.69±0.55a	0.07±0.03a	0.01±0.00a
Ascobolus	0.07±0.02c	0.04±0.01c	0.61±0.15a	0.37±0.11b
Candida	1.00±0.27a	0.13±0.03b	0.17±0.03b	0.14±0.03b
Lundqvistomyces	0.85±0.17a	0.29±0.26b	0.13±0.04b	0.10±0.03b
Thermomyces	0.73±0.44a	0.10±0.7b	0.07±0.02b	0.02±0.01b
Arthrobotrys	0.31±0.11a	0.14±0.09a	0.03±0.01a	0.01±0.00a
Rozellomycota_gen_Incertae_sedis	0.36±0.18a	0.03±0.03c	0.09±0.04b	0.16±0.02b
Fungi_gen_Incertae_sedis	0.47±0.40a	0.11±0.04a	0.04±0.02a	0.10±0.17a
Pseudogymnoascus	0.62±0.39a	0.10±0.07b	0.19±0.11b	0.09±0.06b

Note: Values represent the mean ± SE (n = 3); Values marked with different letters within a row are significantly different according to Tukey's test ($p < 0.05$).
